# MetaNetter 2: A Cytoscape plugin for ab initio network analysis and metabolite feature classification

**DOI:** 10.1016/j.jchromb.2017.08.015

**Published:** 2017-12-15

**Authors:** K.E.V. Burgess, Y. Borutzki, N. Rankin, R. Daly, F. Jourdan

**Affiliations:** a Glasgow Polyomics, University of Glasgow, Glasgow, United Kingdom; b Toxalim (Research Centre in Food Toxicology), Université de Toulouse, INRA, ENVT, INP-Purpan, UPS, Toulouse, France; c Institute of Cardiovascular and Medical Sciences, University of Glasgow, Glasgow, United Kingdom; d Institute of Infection, Immunity and Inflammation, University of Glasgow, Glasgow, United Kingdom

**Keywords:** MetaNetter, Ab-initio network, Mass spectrometry, Metabolomics

## Abstract

Metabolomics frequently relies on the use of high resolution mass spectrometry data. Classification and filtering of this data remain a challenging task due to the plethora of complex mass spectral artefacts, chemical noise, adducts and fragmentation that occur during ionisation and analysis. Additionally, the relationships between detected compounds can provide a wealth of information about the nature of the samples and the biochemistry that gave rise to them.

We present a biochemical networking tool: MetaNetter 2 that is based on the original MetaNetter, a Cytoscape plugin that creates ab initio networks. The new version supports two major improvements: the generation of adduct networks and the creation of tables that map adduct or transformation patterns across multiple samples, providing a readout of compound relationships.

We have applied this tool to the analysis of adduct patterns in the same sample separated under two different chromatographies, allowing inferences to be made about the effect of different buffer conditions on adduct detection, and the application of the chemical transformation analysis to both a single fragmentation analysis and an all-ions fragmentation dataset.

Finally, we present an analysis of a dataset derived from anaerobic and aerobic growth of the organism *Staphylococcus aureus* demonstrating the utility of the tool for biological analysis.

## Highlights

• An update to the ab-initio network construction tool MetaNetter has been produced.
• The tool creates networks of masses from high resolution mass spectrometry data.
• The new plugin provides both chemical transformation and adduct mapping.
• Tables mapping adduct and transform counts across samples can be generated.
• Retention time windows are supported for both adduct and transform network generation.

## 1 Introduction

Metabolomics is rapidly becoming a standard tool for ‘omics’ research. The application of high-resolution mass spectrometry systems such as the Orbitrap [1] and high resolution Q-ToFs [2] allow the generation of rich datasets with accurate mass information that allows substantial inference on the metabolite composition of a mixture to be obtained.

In complex mixtures, features detected using high resolution MS are often interpreted independently. These features are nevertheless related to each other since they may be involved in the same biochemical reactions (one being the substrate and the other the product). They also may be technologically connected due to ionisation modes (e.g. fragments, adducts). When annotating, it is important to take into account this information since it can provide valuable clues on the molecular nature of features. A common way to elucidate adducts and fragments consists in clustering peaks related to the same original compound [3]. By taking biochemistry into account, it is possible to exploit the constraints implied by the metabolic network (the union of all biochemical reactions which can occur in a metabolic network) [4]. To develop this network topology it is first necessary to establish a correspondence (mapping) between features and metabolites in the network. The overlap between features and metabolites in the network, however is far from satisfying, meaning that not all the features can be mapped in these networks. One of the reasons is that metabolic networks are inferred from genomic information and common knowledge on metabolism which can be incomplete. The other reason is that some metabolites like lipids are represented in the network by class compounds (e.g. “a sphingolipid”).

To analyse all features in a network perspective, Breitling et al. [5] proposed to build “ab initio” networks based on mass differences. For each pair of features in the network, if the mass difference between them is equal (up to few ppm) to the mass difference of a biochemical reaction, then these two features can be connected. Following this work, we proposed software (a Cytoscape plugin) called MetaNetter [6] allowing this network reconstruction to be performed.

The MetaNetter plugin for Cytoscape 2 provided the capability to perform ab initio network prediction and was in the top 50 downloaded apps. Cytoscape is a powerful software package for displaying and manipulating networks. The original MetaNetter relied on a configurable list of potential chemical transforms. When provided with a list of accurate masses and a mass tolerance to take the inherent noise affecting mass information into account, MetaNetter produces a graph specifying the individual masses as nodes in the network, where predicted transformations are depicted as edges as described in the original paper. This first release of the plugin is not compatible with the new version of Cytoscape (3.0). Moreover it only took into account the biochemical relationships between features and not adducts. This last functionality is important for metabolite annotation as will be shown in this article.

The new version has been substantially rewritten to adhere to the model provided by the latest version of Cytoscape (3.0). An adduct matching tool has been provided alongside the transformations method, allowing annotation of links between nodes that potentially derive from non-proton adducts. Additionally, retention time restriction has been added, allowing matches (predominantly for adduct matching) to be only allowed within a user-definable time window.

The original version of MetaNetter has also been successfully used to map features across multiple samples, allowing the chemical transformations in different states and under different experimental conditions to be observed, for example analysing the chemical relationships within dissolved organic matter from samples at the ocean surface compared to the deep ocean [7]. This new version also computes frequencies of each mass difference and adducts at a given threshold, generating a table which can highlight the overrepresentation of particular transformations, allowing this type of analysis to be performed rapidly and easily.

## 2 Methods and materials

### 2.1 Software

The MetaNetter 2 plugin was written in Java 1.8 using the MAVEN framework for compilation. MetaNetter 2 is an OSGI module compliant with the Cytoscape 3.0 application. MetaNetter 2 is available in the Cytoscape App Store.

### 2.2 Samples

5 ul of foetal calf serum was extracted using 200 uL ice cold chloroform/methanol/water 1:3:1. The resulting mixture was vortexed for 30 s followed by centrifugation for 5 min at 13,000*g*.

Standards mixes were prepared as described as in [8].

A clinical strain of *S. aureus*, 5817-q14, LHSKBClinical [9] was cultured overnight on Brain Heart Infusion (BHI) agar plates (Oxoid Limited, Basingstoke, UK) in a humidified static incubator at 37 °C. For the preparation of sub-cultures, single colonies were taken and inoculated into BHI broth media (Oxoid) in 1,5 mL reaction tubes (Eppendorff). Aliquots (1 mL) of these liquid cultures were then incubated overnight at 37 °C in a shaking incubator with 180 rpm to ensure a stationary growth phase.These were used for the subsequent preparation of biofilm and planktonic cell cultures. The planktonic cell cultures were grown in a shaking incubator with 180 rpm and 37 °C under aerobic and anaerobic conditions, the biofilm samples on the other hand were grown under aerobic conditions in a static incubator at 37 °C.

Planktonic cell and biofilm cell extracts were obtained following the planktonic and biofilm extraction protocols described in Ref. [10].

### 2.3 LC–MS methodology

10 uL of each cell extract was injected onto an UltiMate 3000 RSLC system (Thermo, UK) and separated using a 4 mm × 150 mm ZIC-pHILIC column (for high pH adduct analysis and fragmentation analysis) or a 4 mm × 150 mm ZIC-HILIC column (for low pH adduct analysis). All gradients and solvents were identical (A: water, B: acetonitrile) save for 20 mM ammonium carbonate, used as an ion pair in buffer A for the pHILIC analysis, and 0.1% formic acid added to buffer A and 0.08% formic acid added to buffer B in the HILIC analysis. Gradients started from 20% A, rising to 80% after 15 min with a step to 95% A for 3 min, followed by 6 min equilibration time.

## 3 Results

Similar to MetaNetter, MetaNetter 2 requires an input file in text format with a list of masses. For retention time matching a column containing these times for each peak must be supplied. For correlation sorting or visual mapping of the nodes by abundance, additional columns may be provided headed with a sample label and containing numerical values for abundances.

Datasets used in this example were derived from bovine serum extracts (used as inter-batch quality control) and a standards mix used primarily for improved annotation [8].

A new addition to the capabilities of MetaNetter is the annotation of adducts in a dataset. This works in a similar manner to the chemical transformations described in the original paper, but is also capable of taking into account di- and trimers of the original molecule, decorated with a variety of adducts, as well as multi-charge adducts. Display of the networks is configurable: the ‘visual mapping’ tab allows the colour and size of nodes to be mapped to either the mass of the compound (blue and small nodes are mapped to low masses, and red, large nodes mapped to high masses), or to the intensity of a mass/node pair in a given sample, with blue being low intensity and red being high. Additionally, both adduct and transformation edges are labelled with the adduct or transformation mapped, and furthermore transformations can be colour coded by type. Furthermore, the matching algorithm can be restricted to search only a specific retention time window for matches. For example adduct matches can be restricted to peaks occurring within a 10 s window.

**Fig. 1:**
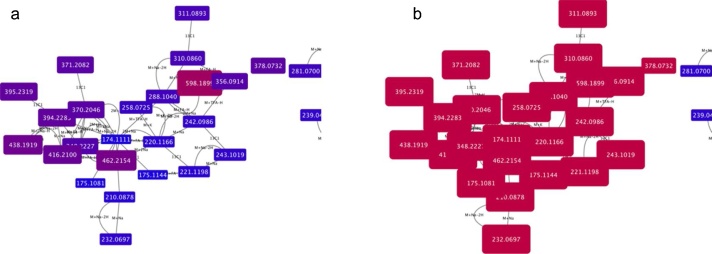
a and b show the adduct pattern for a peak at 174.1111 (centre), corresponding to arginine. Fig. 1a is visually formatted by mass, with red coloured and larger nodes denoting greater masses than blue small nodes. Fig. 1b is visually mapped by retention time, demonstrating that all peaks mapped elute at the same time, towards the end of the run (denoted by the purple colour of the nodes). (For interpretation of the references to colour in this figure legend, the reader is referred to the web version of this article.).

Fig. 1a and b shows the adduct pattern for a peak at 174.1111, corresponding to arginine, from a Leishmania dataset [11]. Fig. 1a is visually formatted by mass, with red coloured and larger nodes being larger than blue small nodes. Fig. 1b is visually mapped by retention time, demonstrating that all peaks mapped elute at the same time, towards the end of the run (denoted by the red colour of the nodes).

**Fig. 2:**
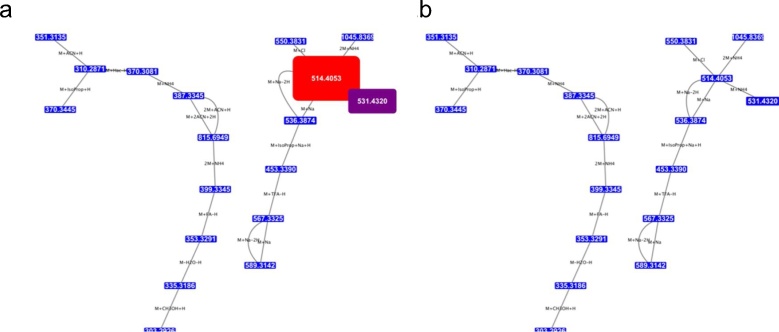
Two ‘base peaks’ and the adduct networks associated with them under a) acidic and b) basic conditions with edges created between nodes with an intensity of over 100,000 counts. Note that the 512 *m*/*z* peak is significantly higher intensity denoted by a larger sized node under acidic conditions than under basic, although the adduct network in both cases is of greater intensity than the threshold.

In this example, bovine serum extracts were analysed in positive mode ionisation under acidic (pH 3.0) and basic (ph 9.0) conditions, using a ZIC-HILIC (Merck Sequant, Sweden) or ZIC-pHILIC (Merck Sequant, Sweden) column buffered with formic acid or ammonium carbonate respectively. Adduct pattern networks (see Fig. 2) were derived from each dataset and a table showing the number of detected adducts in each sample was generated using the Sample Match Table module (Table S1).

Overall, positive ionisation is favoured by a low pH, and consequently the proportion of adducts detected in HILIC is higher than those detected in pHILIC. This is demonstrated in Table S1. The most marked increases in number of detected adducts were those including formate, which was an additive in the HILIC separation but not in the pHILIC. Due to the addition of an ammonium salt in the high-pH pHILIC separation it was expected that more ammonium adducts would be detected, but this is not the case, with the majority detected in the HILIC. It is expected that the effect of any increase in adduct formation is suppressed by the overall effect of high pH on positive mode ionisation.

## 4 Transformations applied to all-ions fragmentation

**Fig. 3:**
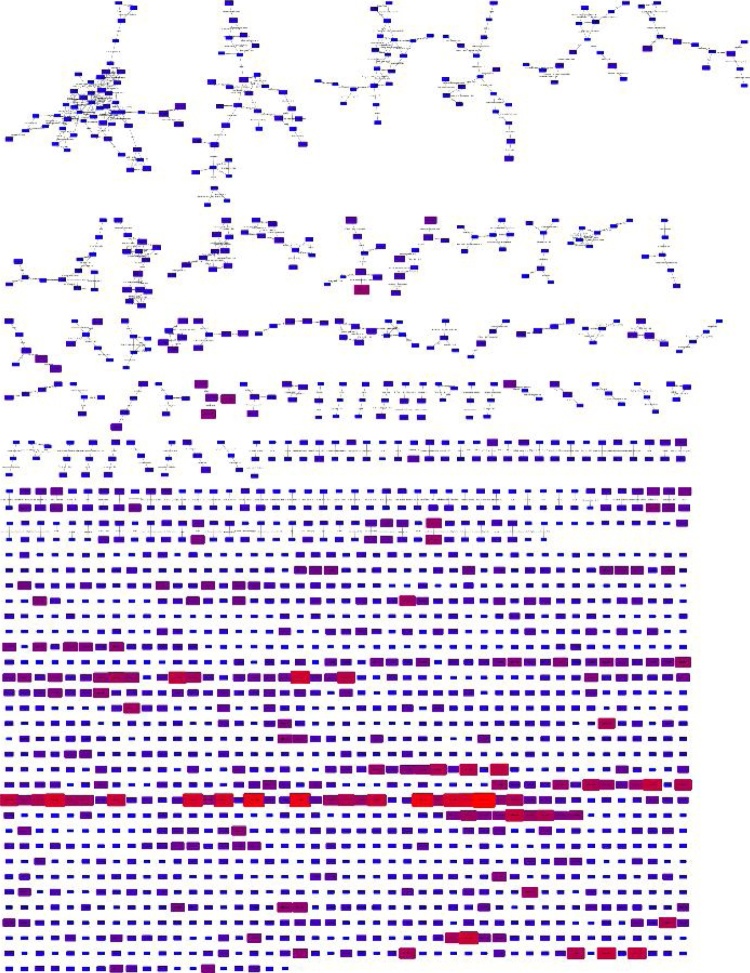
Transformation network combining an all ions fragmentation with the precursor masses from the same sample and applying a time window restriction of 10 s. Note the clusters of interlinked features, potentially fragmentation graphs that can aid in metabolite annotation. Note the row of ‘orphan’ peaks below the interlinked networks – these are due to peaks that are not linked to any other by a common chemical transformation.

Transformation networks were available in the original version of MetaNetter. The new version provides the same functionality but also provides the ability (via the sample match table module) to map types of transformation to particular samples, based on a given threshold, or to determine the average ratio of transformed to untransformed molecules. This allows rapid profiling of the types of transformation detected across multiple samples and may be useful in identification of enzyme functions or the presence of particular chemical modifiers in different samples (see Tables S2 and 1 ). One situation where the utility of the technique is likely to be highly helpful is the annotation of ‘all-ions’ fragmentation data. All-ions fragmentation is the application of collision energy to a sample without isolation of a specific ion first. Usually all ions fragmentation is interleaved with a standard MS scan so that molecular ions can be detected. Most implementations of this technique use correlation analysis to match retention times and peak shapes from precursor scans to fragments in the MSMS scan. MetaNetter’s transformation mapping capability can be used to create networks of interrelated fragments and precursors from MS scans and MSMS scans (see Fig. 3), and the table of transformations allows detection of the types of fragment commonly produced compared to those in the precursor scans (see Table S2). Retention time restriction is used to ensure transformations are only mapped to fragments eluting at the same time. Note, for example, that H_2_O loss and CO loss are extremely frequent in the fragmentation data.

**Table 1:** Transformation table at an intensity threshold of 6 (Log_10_ intensity) of *Staphylococcus* cells under aerobic (Aer) and anaerobic (An) conditions. There is a significant (P value < 0.01) drop in transformation ratios in anaerobic (An) samples for these transformations, denoted by the predominantly green (low) levels of all An samples other than the outlier An_3. (For interpretation of the references to colour in this table legend, the reader is referred to the web version of this article.)

**Fig. 4:**
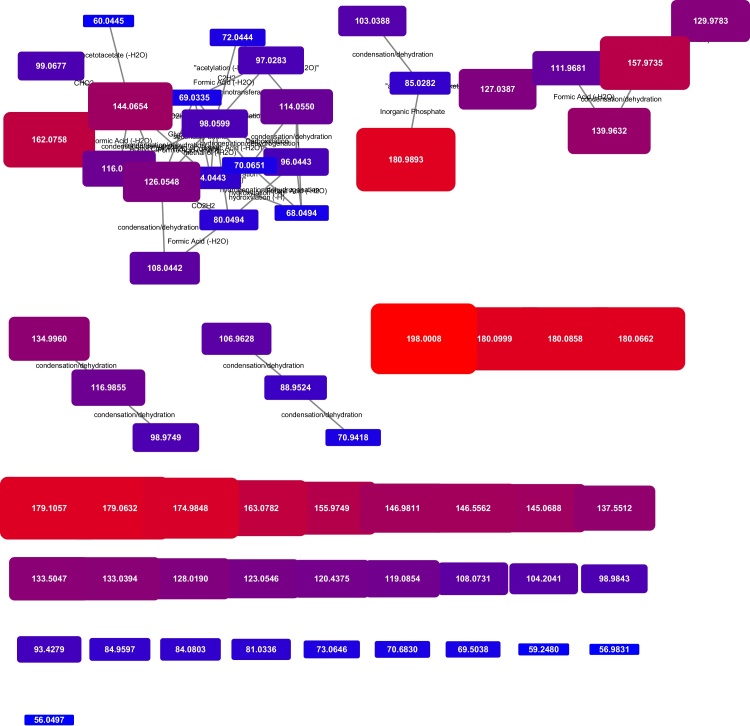
Transformation network from a single selected MSMS spectrum – fragmentation of a 180.09 peak. While several orphan fragments are present, a large cluster of clearly chemically related peaks, linked to the base peak in the spectrum are visible along with four additional peak group clusters.

Individual networks generated from peak lists of selected ions can, of course, be mapped as well, and the data produced is highly useful for annotation. In the example provided in Fig. 4, the transformation network associated with selected fragmentation of a peak with an *m*/*z* ratio of 180.09 is shown, generated from a data-dependant acquisition experiment. The precursor mass is directly linked to a phosphate group loss, while in a second cluster, the base peak (84.0443) is connected to a peak at 98.0599 that is then connected to many other fragments with a variety of chemical transformations. This type of analysis has potential applications in situations where multiple peaks are within a mass spectrometer’s isolation window, contaminating a fragment spectrum, and potentially leading to multiple transformation networks, such as the one described in Fig. 4.

## 5 Analysis of anaerobically and aerobically grown *Staphylococcus aureus*

**Fig. 5:**
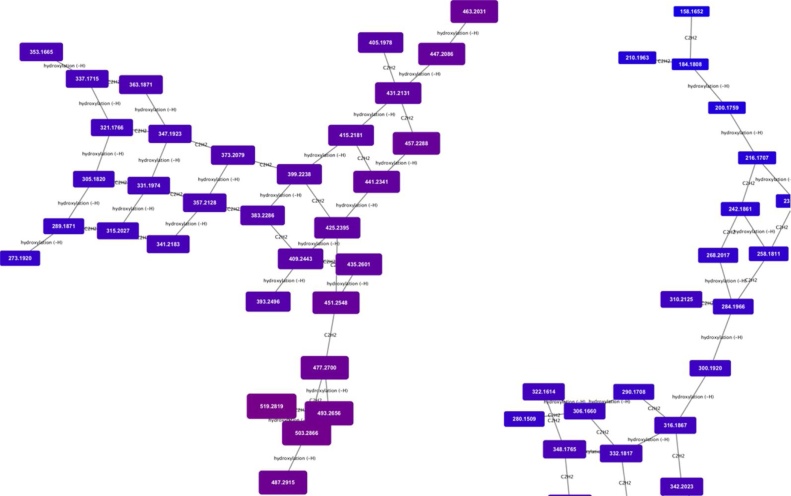
Transformation network focusing on hydroxylation and ethyl group extension. Note that the masses, annotated as lipids and fatty acids, organise into grid patterns linking compounds related by H -> OH and addition or subtraction of C_2_H_2_. Nodes are colour and size coded by mass, and it is possible to see the gradual shift from smaller to larger features across the networks.

**Fig. 6:**
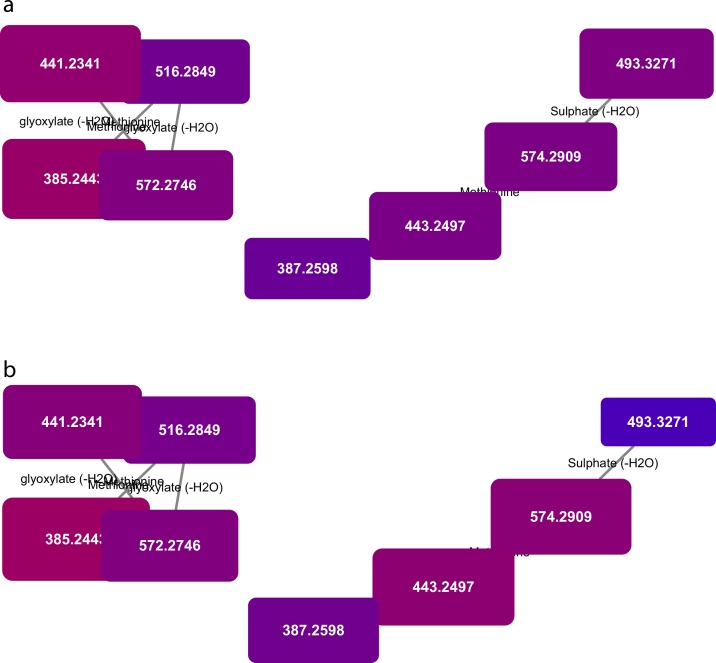
Transformation network specifying only phosphate, methionine, sulphate, diphosphate glucose-*N*-phosphate and glyoxalate transformations, colour and size coded for intensity under aerobic (a) and anaerobic (b) conditions. Note the significant drop in intensity of the compound circled in red under anaerobic conditions. (For interpretation of the references to colour in this figure legend, the reader is referred to the web version of this article.).

To demonstrate the application to a biological sample, we applied the MetaNetter 2 app to a study of the pathogenic, Gram-positive organism *Staphylococcus aureus* grown under aerobic and anaerobic conditions. While not a substitute for a statistically driven untargeted analysis, MetaNetter 2 provides a visual way of looking at properties of the dataset that can provide insights into bulk properties. For example, Fig. 5 shows maps of the predicted lipids, connected via hydroxylation or chain length transformations. It is possible to see the interrelationships between detected compounds visually, as well as provide a visual guide to their relative quantitation. An example of this is given in Fig. 6, where the selected transformations are phosphate, methionine, sulphate, diphosphate glucose-*N*-phosphate and glyoxalate. It is easy to pick out differences from the aerobically grown samples than the anaerobic samples using the table generation function (shown in Table 1), which can produce a table of the average ratio of transformed/untransformed features. In this case the P-values for a decrease in sulphate, methionine, inorganic phosphate and glucose *N*-phosphate transformations under anaerobic conditions are significant (P < 0.01), while glyoxalate transformations are more prevalent (P < 0.01).

## 6 Discussion

Several other methods exist for graph presentation of mass spectrometry data. Software such as Pathos [12] and MassTrix [13] map detected masses on to KEGG pathways. Others like MetExplore [14] can use other databases than KEGG like genome scale networks provided in SBML formats. Nevertheless, both approaches rely only on metabolites belonging to the network and thus don't cover all the features generated in MS experiments. MetScape also allows mapping metabolomics data to these databases for Human (KEGG and EHMN) and add the ability to add correlation edges when semi-quantitative data are available. Other tools like Global Natural Products Social Molecular Networking (GNPS) [15] will generate molecular networks that can be loaded in Cytoscape. These networks are quite useful for identification purposes since they will connect similar MS/MS spectra. This approach is different from the one implemented in MetExplore2 [14] since this method is based on mass differences.

Mummichog is the implementation of an approach aiming at performing metabolomics functional analysis by including features that are not necessarily identified. Mass difference is used to take into account adducts and confirm module or pathways enrichment. Nevertheless, mass difference is not used to reconstruct potential *ab initio* cascades of biochemical transformations in contrary to MetaNetter 2. One limitation of MetaNetter 2 is that it presupposes the existence (at some intensity) of an H^+^ or H^−^ ion that other adducts can be related to. Recent advances to adduct pattern matching [16] are a target for addition to the MetaNetter software in future.

## 7 Conclusion

MetaNetter 2 provides a suite of expanded tools for de-novo network generation that provide unique capabilities to those seeking to annotate mass spectrometry data. The ability to map transformations and adducts across multiple samples to annotate the *types* of transforms and adducts detected on a per sample basis allows rapid screening for chemical and physicochemical modifications.

The utility of MetaNetter 2 has been demonstrated in three usage cases: evaluating the adduct patterns arising from different buffer compositions, analysing the chemical transformations inherent in collisionally induced fragmentation patterns, and an application to *Staphylococcus aureus* grown under aerobic and anaerobic conditions. These cases illustrate only three of the many applications that the software can support and we envision novel applications in the field of metabolite identification to be discovered as usage of the tool expands.
