# Supplementary material for: MetaNetter 2: A Cytoscape plugin for ab initio network analysis and metabolite feature classification
Source: J Chromatogr B Analyt Technol Biomed Life Sci. 2017 Dec 15;1071:68–74. doi: 10.1016/j.jchromb.2017.08.015 (PMC5726607; doi:10.1016/j.jchromb.2017.08.015)
Supplement: Supplementary file 1 [file mmc1.docx]

Supplementary Tables:

| Label | Formula | Mass | Charge | pHILIC1 | pHILIC2 | pHILIC3 | HILIC1 | HILIC2 | HILIC3 |
| --- | --- | --- | --- | --- | --- | --- | --- | --- | --- |
| M+3H | M+3H | 0.0000 | 3+ | 9 | 8 | 9 | 7 | 7 | 7 |
| M+2H+Na | M+2H+Na | 7.3273 | 3+ |  |  |  | 1 | 1 | 1 |
| M+H+2Na | M+H+2Na | 14.7589 | 3+ |  |  |  | 1 | 1 | 1 |
| M+3Na | M+3Na | 21.9819 | 3+ | 3 | 3 | 2 | 2 | 2 | 3 |
| M+2H | M+2H | 0.0000 | 2+ | 18 | 19 | 19 | 29 | 27 | 28 |
| M+H+NH4 | M+H+NH4 | 8.5133 | 2+ | 1 | 1 | 1 |  |  |  |
| M+H+Na | M+H+Na | 10.9910 | 2+ | 6 | 6 | 6 | 13 | 13 | 12 |
| M+H+K | M+H+K | 18.9779 | 2+ | 3 | 3 | 3 | 7 | 7 | 7 |
| M+ACN+2H | M+ACN+2H | 20.5133 | 2+ | 2 | 2 | 1 | 1 | 1 | 1 |
| M+2Na | M+2Na | 21.9819 | 2+ | 7 | 7 | 6 | 15 | 14 | 14 |
| M+2ACN+2H | M+2ACN+2H | 41.0265 | 2+ | 9 | 8 | 7 | 12 | 12 | 12 |
| M+3ACN+2H | M+3ACN+2H | 61.5398 | 2+ |  |  |  |  |  |  |
| M+NH4 | M+NH4 | 17.0265 | + | 135 | 137 | 133 | 156 | 154 | 151 |
| M+Na | M+Na | 21.9819 | + | 97 | 93 | 93 | 102 | 102 | 101 |
| M+CH3OH+H | M+CH3OH+H | 32.0262 | + | 99 | 103 | 104 | 152 | 147 | 148 |
| M+K | M+K | 37.9559 | + | 16 | 16 | 15 | 38 | 37 | 38 |
| M+ACN+H | M+ACN+H | 41.0265 | + | 94 | 92 | 95 | 171 | 168 | 168 |
| M+2Na-H | M+2Na-H | 43.9639 | + | 32 | 33 | 33 | 22 | 23 | 23 |
| M+IsoProp+H | M+IsoProp+H | 60.0581 | + | 14 | 14 | 12 | 22 | 23 | 23 |
| M+ACN+Na | M+ACN+Na | 63.0085 | + | 23 | 23 | 22 | 20 | 19 | 18 |
| M+2K-H | M+2K-H | 75.9118 | + | 8 | 9 | 9 | 12 | 12 | 11 |
| M+DMSO+H | M+DMSO+H | 78.0139 | + | 8 | 9 | 8 | 38 | 37 | 36 |
| M+2ACN+H | M+2ACN+H | 82.0531 | + | 21 | 21 | 21 | 48 | 48 | 49 |
| M+IsoProp+Na+H | M+IsoProp+Na+H | 83.0478 | + | 13 | 12 | 12 | 18 | 17 | 17 |
| 2M+H | 2M+H | 0.0000 | + | 18 | 19 | 19 | 29 | 27 | 28 |
| 2M+NH4 | 2M+NH4 | 17.0265 | + | 10 | 10 | 9 | 26 | 24 | 24 |
| 2M+Na | 2M+Na | 21.9819 | + | 7 | 7 | 6 | 15 | 14 | 14 |
| 2M+K | 2M+K | 37.9559 | + | 4 | 4 | 4 | 8 | 8 | 8 |
| 2M+ACN+H | 2M+ACN+H | 41.0265 | + | 9 | 8 | 7 | 12 | 12 | 12 |
| 2M+ACN+Na | 2M+ACN+Na | 63.0085 | + | 3 | 3 | 3 | 8 | 8 | 8 |
| M-3H | M-3H | 0.0000 | 3- | 9 | 8 | 9 | 7 | 7 | 7 |
| M-2H | M-2H | 0.0000 | 2- | 18 | 19 | 19 | 29 | 27 | 28 |
| M-H2O-H | M-H2O-H | -18.0111 | - | 13 | 13 | 13 | 22 | 22 | 22 |
| M+Na-2H | M+Na-2H | 21.9819 | - | 97 | 93 | 93 | 102 | 102 | 101 |
| M+Cl | M+Cl | 35.9767 | - | 20 | 19 | 21 | 34 | 33 | 34 |
| M+K-2H | M+K-2H | 37.9559 | - | 16 | 16 | 15 | 38 | 37 | 38 |
| M+FA-H | M+FA-H | 46.0055 | - | 102 | 102 | 107 | 150 | 149 | 148 |
| M+Hac-H | M+Hac-H | 60.0211 | - | 98 | 98 | 97 | 133 | 133 | 130 |
| M+Br | M+Br | 79.9262 | - | 3 | 3 | 3 | 7 | 7 | 7 |
| M+TFA-H | M+TFA-H | 113.9929 | - | 13 | 13 | 12 | 19 | 18 | 19 |
| 2M-H | 2M-H | 0.0000 | - | 18 | 19 | 19 | 29 | 27 | 28 |
| 2M+FA-H | 2M+FA-H | 46.0055 | - | 8 | 9 | 8 | 35 | 36 | 36 |
| 2M+Hac-H | 2M+Hac-H | 60.0211 | - | 4 | 4 | 4 | 17 | 17 | 17 |
| 3M-H | 3M-H | 0.0000 | - | 9 | 8 | 9 | 7 | 7 | 7 |

Table S1: Adduct network. The dataset consists of 3 technical replicates of foetal bovine serum samples analysed under basic and acidic conditions. Data shown is from positive ionisation mode. Note that overall, there are more adducts detected in the HILIC samples. This is likely to be due to the overall preference for ionisation of positively charged adducts in postitive mode. This is especially notable in formate adducts (from an average of 100 to 150 in HILIC), which are likely also due to formate in the buffer.

| Label | Formula | Mass | MS_1 | MS_2 | Frag_1 | Frag_2 |
| --- | --- | --- | --- | --- | --- | --- |
| Aminotransferase | | 1.031634 | 3 | 3 | 4 | 4 |
| hydrogenation/dehydrogenation | H2 | 2.01565 | 5 | 4 | 19 | 19 |
| tertiary amine | N | 14.00307 |  |  | 6 | 6 |
| methanol (-H2O) | CH2 | 14.01565 | 5 | 7 | 21 | 21 |
| secondary amine | NH | 15.0109 | 1 |  | 6 | 6 |
| hydroxylation (-H) | O | 15.99491 | 14 | 12 | 22 | 22 |
| primary amine | NH2 | 16.01872 |  |  | 5 | 5 |
| condensation/dehydration | H2O | 18.01056 | 28 | 27 | 66 | 68 |
| C2H2 | C2H2 | 26.01565 | 2 | 1 | 13 | 13 |
| Formic Acid (-H2O) | CO | 27.99491 | 19 | 19 | 51 | 51 |
| "Ethanol (-H2O), ethyl addition (-H2O)" | C2H4 | 28.0313 | 1 | 1 | 6 | 6 |
| Inorganic Phosphate | HPO4 | 95.96125 |  |  |  |  |
| "acetylation (-H2O), ketol group (-H2O)" | C2H2O | 42.01056 | 14 | 10 | 14 | 13 |
| Arg>Orn | CH2N2 | 42.0218 | 2 | 2 | 3 | 3 |
| Ethanolamine (-H2O) | C2H4N | 42.03437 | 1 | 1 | 12 | 13 |
| Carboxylation | CO2 | 43.98983 | 7 | 8 | 6 | 6 |
| carbamoyl P transfer (-H2PO4) | CH2ON | 44.01364 | 1 | 1 | 1 | 1 |
| CHO2 | CHO2 | 44.99765 | 1 |  | 1 | 1 |
| CO2H2 | CO2H2 | 46.00548 | 30 | 32 | 41 | 43 |
| glyoxylate (-H2O) | C2O2 | 55.98983 | 6 | 6 | 26 | 25 |
| PolyamineReactionA | | 56.03745 | 1 | 1 |  |  |
| Glycine | C2H3NO | 57.02146 | 5 | 3 | 7 | 7 |
| acetone (-H) | C3H5O | 57.03404 |  |  |  |  |
| Polyamine Synthase | C3H7N | 57.05785 | 3 | 3 | 2 | 2 |
| acetylation (-H) | C2H3O2 | 59.0133 | 1 |  | 2 | 2 |
| urea addition (-H) | CH3N2O | 59.02454 | 2 | 2 | 1 | 1 |
| Acetyl CoA + H2O (-CoASH) | C2H4O2 | 60.02113 | 7 | 6 | 8 | 9 |
| pyrophosphate | PP | 61.94753 | 1 | 1 |  |  |
| Mevalonate-5-pyrophosphate decarboxylase | CH2O3 | 62.0004 | 3 | 4 | 2 | 2 |
| isoprene addition (-H) | C5H7 | 67.05478 |  |  | 2 | 2 |
| Alanine | C3H5NO | 71.03711 | 4 | 3 | 4 | 4 |
| sulfate (-H2O) | SO3 | 79.95682 | 1 | 1 | 2 | 2 |
| phosphate | HPO3 | 79.96633 |  |  | 2 | 2 |
| Sulphate (-H2O) | HSO3 | 80.96464 | 1 | 1 | 1 | 1 |
| acetotacetate (-H2O) | C4H4O2 | 84.02113 | 2 | 2 | 2 | 3 |
| malonyl group (-H2O) | C3H2O3 | 86.00039 |  |  |  |  |
| Choline (-H2O) | C5H12N | 86.09697 |  |  |  |  |
| Serine | C3H5NO2 | 87.03203 | 9 | 10 | 8 | 8 |
| Proline | C5H7NO | 97.05276 | 2 | 2 | 2 | 2 |
| Valine | C5H9NO | 99.06841 | 3 | 3 | 1 | 1 |
| Threonine | C4H7NO2 | 101.0477 |  |  |  |  |
| Erythose (-H2O) | C4H6O3 | 102.0317 | 3 | 3 | 2 | 3 |
| Cysteine | C3H5NOS | 103.0092 |  |  | 1 | 1 |
| cytosine (-H) | C4H4N3O | 110.0354 | 2 | 2 | 1 | 1 |
| uracil (-H) | C4H3N2O2 | 111.0195 |  |  | 1 | 1 |
| "Isoleucine, Leucine" | C6H11NO | 113.0841 | 2 | 2 |  |  |
| Asparagine | C4H6N2O2 | 114.0429 | 5 | 5 | 1 | 1 |
| Ornithine (-H2O) | C5H10N2O | 114.0793 | 2 | 2 |  |  |
| Aspartic Acid | C4H5NO3 | 115.0269 | 2 | 2 | 2 | 2 |
| thymine (-H) | C5H5N2O2 | 125.0351 |  |  |  |  |
| Glutamine | C5H8N2O2 | 128.0586 | 2 | 2 | 3 | 3 |
| Lysine | C6H12N2O | 128.095 | 2 | 2 | 1 | 1 |
| PolyamineReactionB | | 128.1313 |  |  |  |  |
| Glutamic Acid | C5H7NO3 | 129.0426 | 1 | 2 | 1 | 1 |
| Methionine | C5H9NOS | 131.0405 | 2 | 2 | 3 | 2 |
| D-Ribose (-H2O) (ribosylation) | C5H8O4 | 132.0423 | 13 | 13 | 13 | 11 |
| adenine (-H) | C5H4N5 | 134.0467 | 1 | 1 | 1 | 1 |
| Histidine | C6H7N3O | 137.0589 |  |  |  |  |
| Phenylalanine | C9H9NO | 147.0684 | 1 | 1 |  |  |
| guanine (-H) | C5H4N5O | 150.0416 |  |  |  |  |
| Arginine | C6H12N4O | 156.1011 |  |  |  |  |
| diphosphate | H3O6P2 | 160.9405 |  |  | 1 | 1 |
| monosaccharide (-H2O) | C6H10O5 | 162.0528 |  |  | 1 | 1 |
| Tyrosine | C9H9NO2 | 163.0633 | 1 | 1 |  |  |
| DHAP | C3H7O6P | 169.998 | 2 | 2 | 2 | 2 |
| Glucuronic Acid (-H2O) | C6H8O6 | 176.0321 | 1 | 1 | 1 | 1 |
| C6H10O6 | C6H10O6 | 178.0477 |  |  |  |  |
| Tryptophan | C11H10N2O | 186.0793 |  |  |  |  |
| Cystine | C6H10N2O3S2 | 222.0133 |  |  |  |  |
| thymidine (-H2O) | C10H12N2O4 | 224.0797 |  |  |  |  |
| uridine (-H2O) | C9H10N2O5 | 226.059 |  |  |  |  |
| biotinyl (-H2O) | C10H14N2O2S | 226.0776 | 1 | 1 |  |  |
| pyridoxal phosphate (-H2O) | C8H8NO5P | 229.014 |  |  |  |  |
| palmitoylation (-H2O) | C16H30O | 238.2297 |  |  |  |  |
| glucose-N-Phosphate (-H2O) | C6H11O8P | 242.0192 |  |  |  |  |
| biotinyl (-H) | C10H15N2O3S | 243.0803 |  |  |  |  |
| adenosine (-H2O) | C10H11N5O3 | 249.0862 | 1 | 1 |  |  |
| guanosine (-H2O) | C10H11N5O4 | 265.0811 |  |  |  |  |
| Sphingosine (-H2O) | C18H35NO | 281.2719 |  |  |  |  |
| glutathione (-H2O) | C10H15N3O5S | 289.0732 |  |  |  |  |
| thymidine 5' monophosphate (-H2O) | C10H13N2O7P | 304.046 |  |  |  |  |
| cytidine 5' monophsophate (-H2O) | C9H12N3O7P | 305.0413 | 1 | 1 |  |  |
| uridine 5' monophosphate (-H2O) | C9H11N2O8P | 306.0253 |  |  |  |  |
| "2-C-methyl-D-erythritol 2,4-cyclodiphosphate synthase " | C9H14N3O8P | 323.0519 |  |  |  |  |
| "Adenosine 5'monophosphate (-H2O), adenylate (-H20)" | C10H12N5O6P | 329.0525 | 1 | 1 | 1 | 1 |
| disaccharide (-H2O) | C12H20O11 | 340.1006 |  |  |  |  |
| Guanosine 5- monophosphate (-H2O) | C10H12N5O7P | 345.0474 |  |  |  |  |
| deoxythymidine 5' diphosphate (-H2O) | C10H14N2O10P2 | 384.0124 |  |  |  |  |
| cytidine 5' diphosphate (-H2O) | C9H13N3O10P2 | 385.0076 |  |  |  |  |
| uridine 5' diphosphate (-H2O) | C9H12N2O11P2 | 385.9916 |  |  |  |  |
| Adenosine 5'-diphosphate (-H2O) | C10H13N5O9P2 | 409.0189 |  |  |  |  |
| PolyamineReactionC | | 417.2046 |  |  |  |  |
| Guanosine 5- diphosphate (-H2O) | C10H13N5O10P2 | 425.0138 |  |  |  |  |
| trisaccharide (-H2O) | C18H30O15 | 486.1585 |  |  |  |  |
| CoASH (-h2o -C4H4O5) | C17H30N7O10P3S | 617.0988 |  |  |  |  |
| CoASH (-glycince) | C19H31N6O14P3S | 692.0832 |  |  |  |  |
| Trypanothione (-H2O) | C27H47N9O9S2 | 705.2938 |  |  |  |  |
| CoASH (-H2CO2) | C20H34N7O14P3S | 721.1097 |  |  |  |  |
| co-enzyme A (-H2O) | C21H34N7O15P3S | 749.1047 |  |  |  |  |
| Succinyl-CoA synthetase | | 751.1203 |  |  |  |  |
| Thyroxine (-H2O) | C15H9I4NO3 | 758.6762 |  |  |  |  |
| HMG-CoA reductase | C21H32N7O16P3S | 763.0839 |  |  |  |  |
| co-enzyme A (-H) | C21H35N7O16P3S | 766.1074 |  |  |  |  |

Table S2: Transformations table demonstrating the most common types of structural change on fragmentation in a given dataset.
